# A mouthful - airway matters in intraoperative neuromonitoring in auditory brainstem implant surgery for the pediatric patient: a case series

**DOI:** 10.1186/s12871-018-0628-z

**Published:** 2018-11-07

**Authors:** Charis Khoo, A. H. NurHafiizhoh, Angela Tan, Tracy Tan, Hwan Ing Hee

**Affiliations:** 1 Department of Paediatric Anaesthesia, KK Women’s and Children’s Hospital, 100 Bukit Timah Road, Singapore, 229899 Singapore; 2 DUKES NUS Medical School, Singapore, Singapore

**Keywords:** Child, Auditory brain stem implants, Intraoperative neurophysiological monitoring, Airway management, Evoked potentials, motor

## Abstract

**Background:**

The Auditory brainstem implant (ABI) is a new surgical option for hearing impaired children. Intraoperative neurophysiology monitoring includes brainstem mapping of cranial nerve (CN) IX, X, XI, XII and their motor nuclei, and corticobulbar tract motor-evoked potential. These require laryngeal electrodes and intra-oral pins, posing a challenge to airway management especially in the pediatric airway, where specialized electromyogram (EMG) tracheal tubes are not available.

Challenges include determining the optimum position on the endotracheal tube (ETT) in which to place laryngeal electrode, and the increase in external diameter of ETT contributed by the wrapping the electrode around the shaft of ETT; this may necessitate downsizing of the tracheal tube. An appropriate size ETT minimizes displacement, which in turn can affect electrode contact with the vocal cords. Finally, a small thus crowded pediatric airway makes for difficult visualization during placement of intraoral neuromonitoring electrodes. The use of a videolaryngoscope helps determine optimum electrode placement.

**Case presentation:**

We describe intraoperative neurophysiology monitoring and airway management for the first two ABI procedures in Singapore, conducted for children with congenitally absent cochlear nerves.

**Conclusion:**

Neurophysiology cranial nerve IX, X, XII monitoring in the ABI procedure requires intraoral placement of electrodes. Care should be exercised during placement and removal. Vagus nerve monitoring in children requires attention to tube preparation, and consideration should be given to avoidance of airway topicalization.

## Background

The auditory brainstem implant is a novel treatment for hearing impairment [1]. ABI has been used for children in Europe over a decade, while clinical trials for children with non Neurofibromatosis-2 conditions was approved in United states in 2013 [2].

This series describes challenges in airway management and intraoperative neurophysiology monitoring for the first two cases in Singapore. We hope that this description will facilitate selection and preparation of tracheal tubes. This will conserve resources, and importantly, minimize airway trauma.

## Case presentation

Informed consent was obtained from parents of both patients.

### Case presentation 1

A 7-year-old, 18 kg, ASA-PS 1 boy, with congenital bilateral sensorineural deafness and failed right cochlear implant. He underwent a Magnetic Resonance Imaging (MRI) before transfer to operating theatre for ABI insertion. During the MRI, his airway was secured with a size 5.5 mm internal-diameter (I.D.) uncuffed ETT.

Planned intraoperative neurophysiology monitoring included brainstem auditory sensory evoked potentials, brainstem mapping of CN IX, X, XI, XII and their motor nuclei, and corticobulbar tract motor-evoked potential (MEP). Lead placement for CN IX, X, XI, XII was performed by the anesthesia team.

**Fig. 1:**
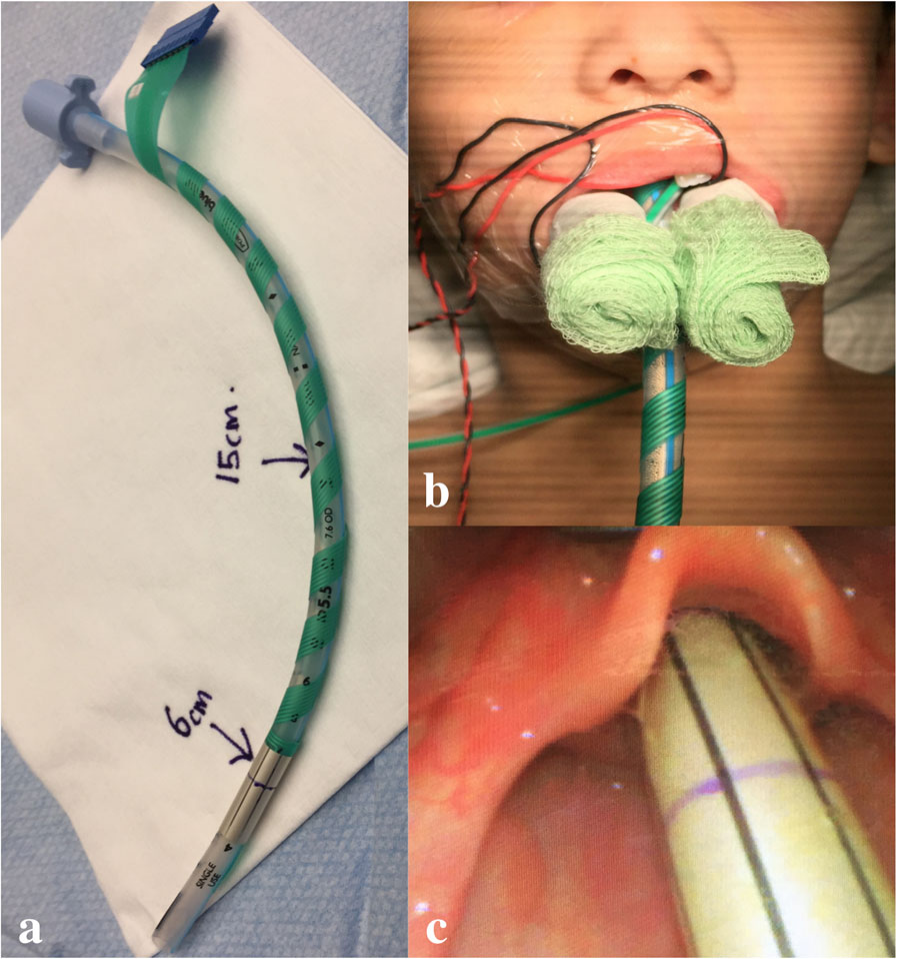
**a** CN X electrode (sensor in silver). **b** Bilateral bite blocks minimise displacement. c Electrode displacement despite careful positioning (end procedure)

CN X monitoring (Fig. 1a) was performed using a 32 mm by 29 mm laryngeal electrode (Inomed, Emmendingen, Germany). To identify optimal electrode placement on the tracheal tube, the patient was positioned as intended for surgery (right lateral), and the depth of ETT corresponding to the laryngeal inlet was identified using C-MAC Laryngoscope (KARL STORZ, Deutschland). This corresponded to 6 cm at vocal cords (15 cm at lips). A new #5.5 uncuffed ETT was then prepared with the laryngeal electrodes (Fig. 1a) and the child re-intubated, in keeping with the measurements. Using C-MAC, the pin electrode for CN IX was placed on the ipsilateral soft palate; electrodes for CN XII were placed on the anterior tongue. Rolled up gauzes were placed on either side of the tracheal tube (Fig. 1b) to stabilize the tracheal tube, and as a bite block.

Total intravenous anesthesia (TIVA) included propofol (Paedsfusor Target Control Infusion, target plasma concentration 3.5–5.0 mcg/ml) [3], remifentanil (0.08–0.3 mcg/kg/min) and ketamine (0.2–0.33 mcg/kg/min). Analgesia included intravenous paracetamol 15 mg/kg, morphine 2.5 mg and local anesthetic infiltration. Anti-emetics (dexamethasone 0.2 mg/kg, ondansetron 0.1 mg/kg) were administered. Surgery was uneventful except for 3 episodes of transient bradycardia (40/min). Duration of procedure, including MRI was 9 h.

### Case presentation 2

A 6-year old girl, 18.9 kg, ASA-PS 1, with congenitally bilaterally absent cochlear nerves scheduled for a right ABI implant.

A #2.5 laryngeal mask was used during pre-operative MRI, and changed to a #5.0 mm-internal-diameter microcuff tube for surgery.

The CN X electrode was wrapped above the ETT cuff at the level of the intubation depth marker (4 cm). The patient was then intubated using a C-MAC Pocket Monitor (KARL STORZ, Deutschland), the ETT secured at the depth of 4 cm at vocal cords, 12 cm at lips. Subsequently, the anesthetist placed intraoral electrodes, and the patient was then positioned for surgery.

Total intravenous anesthesia (TIVA) was conducted with propofol and remifentanil. Analgesia included paracetamol, morphine, and local anesthetic infiltration. Dual antiemetics, dexamethasone and ondansetron were administered. The procedure was uneventful.

In both, laryngoscopy at the end of procedure revealed cranially displaced tracheal tubes (Fig. 1c). Both patients experienced nausea with poor appetite for 2 days despite anti-emetics.

## Discussion and conclusions

Intraoperative neuromuscular monitoring is necessary to prevent iatrogenic injury. Brainstem mapping of CN IX, X, XI, XII and their motor nuclei, and corticobulbar tract motor-evoked potentials help monitor function of cranial nerves, and integrity of ascending sensory tracts and descending motor pathways [4]. Brainstem auditory evoked responses confirm successful device implantation.

Lead placement for cranial nerves IX, X, XI, XII involves the anesthetist and presents challenges for airway management.

**Fig. 2:**
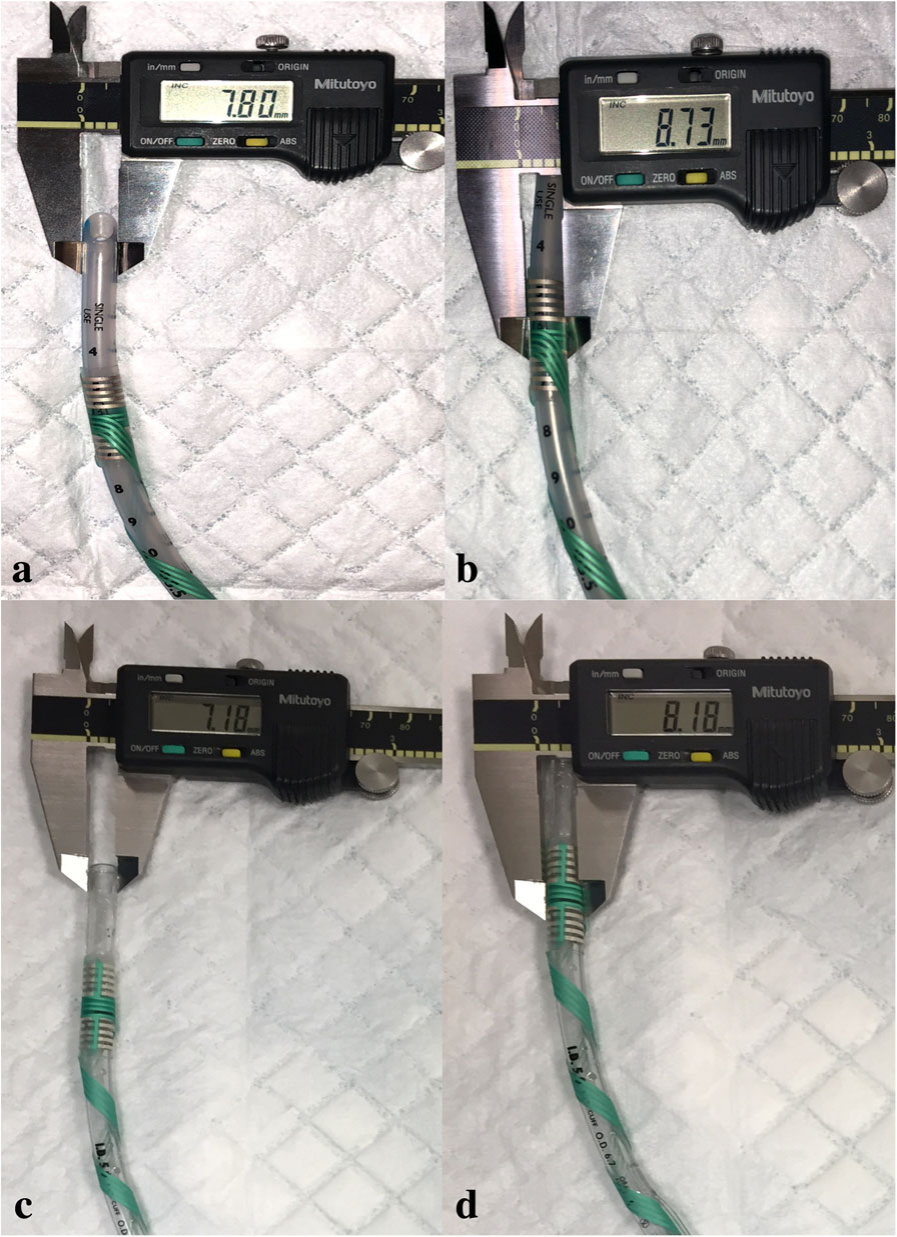
Increased external diameter due to electrode. A and b is demonstrated on a #5.5 uncuffed ETT, c and d is demonstrated on a #5.0 micrcuff ETT

**Fig. 3:**
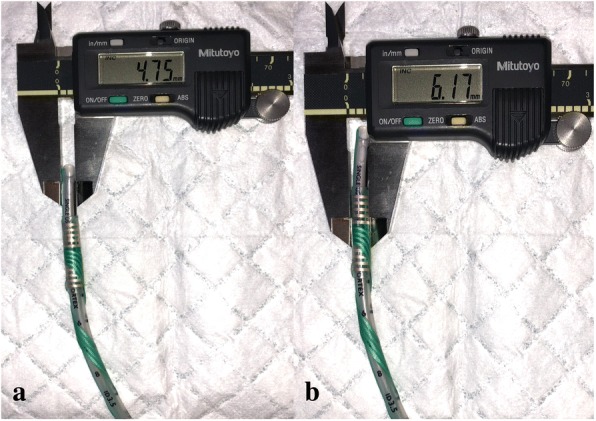
Same electrode demonstrated on a #3.5 ETT. There is a larger increase in external diameter due to overlapping of the wrappings (~ 1.4 mm), compared to its application on a larger ETT (c.f. Fig. 2, where increase in external diameter is ~ 1 mm)

Monitoring of CN X in children may require adhesive laryngeal electrodes when specialized pediatric EMG tracheal tubes are unavailable. Using these electrodes require pre-determination of optimum tube size and depth. Wrapping these electrodes around tracheal tubes increase external diameter (Fig. 2) and stiffness; prudent ETT size selection is necessary to prevent airway trauma. In addition, when these electrodes are applied to smaller tube sizes to accommodate smaller patients, the bulk of the electrode becomes more apparent relative to the ETT (Fig. 3), further downsizing of tracheal tube should be kept in view. Following our experience from patient 1, a slightly smaller tube was selected for patient 2, the microcuff advantageous because cuff inflation would ensure good seal despite downsizing should a leak arise, and minimize the need for tube change.

A new procedure presents a steep learning curve, not only in technicalities but also in workflow process. Our MRI location is separate and remote from the operating theatre. Definitive airway and electrode placement was conducted post MRI, in the operating theatre, as the electrodes are not MRI compatible. Patient 1 presented with mild rhinorrhea, but appeared otherwise well. We decided to proceed with the case as international expertise was engaged to advise on this first attempt in Singapore. Hence, patient 1 was intubated at MRI for transport to operating theatre, to eliminate en route laryngospasm. For patient 2, a laryngeal mask was chosen first, as a microcuff ETT is a limited and more expensive resource. To date, the workflow has evolved such that the preoperative MRI is conducted on another occasion, to minimize risks associated with transport.

Bite blocks on either side of the tracheal tube prevent bite occlusion and centralize the tube, maintaining optimal contact between electrode and bilateral vocal cords. Positioning the patient in a simulated surgical position first enables best estimation of electrode position on the tracheal tube for CN X monitoring during surgery. Video-laryngoscope aids in optimizing placement of the laryngeal electrode, and in placement of IX pin electrodes in the soft palate. In both cases the ETT had displaced slightly in a cranial fashion by the end of the procedure, and we recommend wrapping the electrode slightly lower with this in mind.

Tracheal intubation was facilitated using remifentanil. Vocal cord topicalization and muscle relaxants were omitted to avoid interference with CN X and MEP monitoring respectively. Where evidence regarding the impact of local anaesthetic topicalization on neuromonitoring integrity appears to be conflicting [5], we feel that this is a potential simple confounder, which can be simply eliminated from the complex equation of troubleshooting loss of signal [6].

Intra-oral CN IX and CN XII pins pose a foreign body aspiration risk. A practice of preoperative huddle and verification process tracks the number of intraoral implants placed. A sign out process at procedure end confirms that all electrodes are removed.

Both children experienced prolonged postoperative nausea and vomiting despite anti-emetics, a larger cohort study is needed to confirm this observation.

In summary, neurophysiology monitoring of cranial nerve IX, X, XII in the ABI procedure requires intraoral placement of electrodes. Care should be exercised during placement and removal. Vagus nerve monitoring in children requires attention to tube preparation. Consideration should be given to avoidance of airway topicalization.

1. Intraoperative neuromuscular monitoring for auditory brainstem implant includes brainstem auditory evoked response, brainstem mapping of CN IX, X, XI, XII and their motor nuclei, and corticobulbar tract motor-evoked potential monitoring.
2. Monitoring of cranial nerve X in children requires placement of laryngeal electrodes around tracheal tube, necessitating pre-determination of optimal tube size and depth – Considerations include downsizing and potential displacement.
3. Avoidance of topical anesthesia to larynx facilitates intraoperative neuromonitoring of motor X nerve.

## Abbreviations

ABI: Auditory Brainstem Implant
CN: Cranial Nerve
EMG: Electromyogram
ETT: Endotracheal tube
LA: Local anesthestic
MEP: Motor Evoked Potential
MRI: Magnetic Resonance Imaging
